# Efficacy and Safety of COX-2 Inhibitors in the Clinical Management of Arthritis: Mini Review

**DOI:** 10.5402/2011/480291

**Published:** 2011-05-17

**Authors:** Sam T. Mathew, Gayathri Devi S, V. V. Prasanth, B. Vinod

**Affiliations:** ^1^Medical Writing Group, Accenture Pharmaceutical Services, Karnataka, Bangalore 560072, India; ^2^Allied Health Sciences, Sikkim Manipal University, Karnataka, Bangalore 560008, India; ^3^Department of Pharmaceutics, Gautham College of Pharmacy, Karnataka, Bangalore 560032, India; ^4^Department of Pharmaceutical Chemistry, Pushpagiri College of Pharmacy, Thiruvalla, Kerala 689101, India

## Abstract

In the clinical management of arthritis, the choice of nonsteroidal anti inflammatory drug (NSAID) remains confusing and controversial. A common practice on the choice of NSAID in clinical management of arthritis is the risk benefit ratio. The main objective of this review is to addresses the main arguments for the pharmacological and clinical use of COX-2 inhibitors in relation to nonselective NSAIDs for the clinical management of arthritis. This review concluded that, both NSAIDs and COX-2 inhibitors are equally effective and are associated with increased risk of GI, renal, and CV, adverse effects. Complete understanding of the patient's comorbid conditions and concomitant medications, coupled with precise monitoring during the treatment, may help to decrease the threat of adverse effects induced by nonselective NSAIDs and selective COX-2 inhibitors.

## 1. Introduction

Arthritis is a complex disorder that comprises more than hundred distinct conditions involving damage to the joints of the body. Many mediators are known to be involved in the pathophysiology and progression of arthritis. These include cartilage-degrading enzymes, cytokines, leukotrienes (LTs), and prostaglandins (PGs). LTs and PGs are produced by the activity of three enzymes–5-lipoxygenase, cyclooxygenase (COX)-1 and COX-2–as part of the arachidonic acid (AA) pathway. PGs have various physiological and pathophysiological effects. PGs produced by COX-1 isoenzyme exert house-keeping functions, including gastric mucosal defense and renal homeostasis, whereas COX-2 synthesizes detrimental PGs which are responsible for inflammation and pain. The activity of COX-2 leads to production of a narrower spectrum of PGs, specifically PGE_2_ and PGI_2_. The vasodilatory properties of these two molecules increase mucus production and reduce acid and pepsin levels in the stomach, thereby protecting the integrity of the gastrointestinal (GI) mucosa [1–4].

The ultimate goal for arthritis treatment is the modification of disease progression, in combination with anti-inflammatory and analgesic efficacy [5–7]. Traditional nonselective, nonsteroidal anti-inflammatory drugs (NSAIDs) have been widely used to relieve the pain and inflammation due to osteoarthritis and rheumatoid arthritis. These drugs possess potent anti-inflammatory, analgesic, and antipyretic activity and are among the most widely used drugs worldwide and represent a mainstay in the treatment of acute and chronic pain [8]. However, numerous reported adverse drug reactions, case-control, and postmarketing surveillance studies have revealed that their use is frequently associated with a relatively high incidence of adverse reactions in the GI tract [9–11]. GI toxicity is clinically important because it has been shown to increase morbidity and mortality rates in patients, particularly in the elderly, with chronic therapy [12–18].

Traditional NSAIDs act by inhibiting both COX-1 and COX-2, thereby blocking the synthesis of PGs. The GI adverse events of NSAIDs are majorly due to the decrease in synthesis of the gastroprotective prostaglandins PGE_2_ and PGI_2, _which are mainly produced by COX-1 [1–4, 10]. To significantly reduce the GI toxicity of NSAIDs, associated with acute and chronic use and to obtain similar or better efficacy, pharmaceutical companies conducted intensive international research which led to the development of COX-2 inhibitors [19, 20]. Due to the great expectation, these drugs were rapidly introduced in the market and gained a remarkable commercial and therapeutic success [19–23]. 

## 2. Safety of Traditional NSAIDs versus COX-2 Inhibitors

A number of clinical trials have been conducted over the past 15 years that generally support the favorable GI side effect profile of COX-2 selective inhibitors. Traditional nonselective NSAIDs vary in their propensity to cause serious GI adverse effects. Ibuprofen is associated with the lowest risk; diclofenac, naproxen, indometacin, and ketoprofen have intermediate risks [24]. It was reported that the point prevalence of ulcers in patients on long-term NSAID treatment is about 20%, and the annual incidence of serious complications from these ulcers is 1–4% [25].

A recently published systematic review investigated the relationship between NSAID use and lower GI outcomes. This study reported an increase in lower GI injury and clinical events with traditional NSAIDs, which was consistent across the heterogeneous collection of trials [26]. Many other systematic reviews and meta-analysis have demonstrated comparatively better GI safety for celecoxib, nimesulide, and etodolac in comparison with the traditional NSAIDs [27–31].

Celecoxib Long-term Arthritis Safety Study (CLASS) and Vioxx Gastrointestinal Outcomes Research Study (VIGOR)—large long-term trials—have been conducted in patients with rheumatoid arthritis and osteoarthritis, both involving more than 8,000 subjects. These studies demonstrated that both celecoxib and valdecoxob significantly reduced the risk of major GI side effects compared to traditional NSAIDs [32, 33]. Similar results were obtained in the Therapeutic Arthritis Research and Gastrointestinal Event Trial (TARGET), conducted on 18,325 patients, comparing lumiracoxib with two NSAIDs, naproxen and ibuprofen [34]. Thus findings of published data reveal that the incidence of adverse GI effects is significantly reduced among patients taking selective COX-2 inhibitors. 

## 3. Efficacy and Safety of COX-2 Inhibitors

Even though the GI toxicity profile of selective COX-2 inhibitors is better than the traditional NSAIDs, current evidences indicate that selective COX-2 inhibitors have important adverse cardiovascular and renal effects. The cardiovascular adverse events of selective COX-2 inhibitors include increased risk for myocardial infarction, stroke, heart failure, and hypertension. The science behind the cardiovascular and renal adverse events is explained in literatures [35–38].

Rofecoxib's potential for adverse cardiovascular events was recognized during the VIGOR trial in which patients with rheumatoid arthritis were randomized to 50 mg of rofecoxib once a day or 500 mg of naproxen twice a day. This study indicated a fourfold increase in the incidence of myocardial infarction in the rofecoxib treatment group compared with the naproxen treatment group [33]. A recent study has shown that long-term etoricoxib use is associated with a risk of thrombotic cardiovascular events comparable with that of diclofenac and with a greater risk of renovascular adverse events [39]. A meta-analysis of published and unpublished tabular data from randomized trials revealed that selective COX-2 inhibitors are associated with a moderate increase in the risk of vascular events (relative risk, 1.42; 95% CI, 1.13 to 1.76), as are high-dose regimens of ibuprofen and diclofenac, but high-dose naproxen is not associated with such an excess [40]. High cardiovascular risk associated with sulphone COX-2 inhibitors such as rofecoxib and etoricoxib, as observed in recent clinical trials can be explained by the dose dependent pro-oxidant activity of these classes of drugs in human plasma samples and isolated low-density lipoprotein [41, 42].

Several randomized controlled clinical trials in patients with rheumatoid arthritis or osteoarthritis have demonstrated that COX-2 inhibitors are no more effective than traditional NSAIDs or in other words, have similar efficacy as traditional NSAIDs [27, 43–46]. 

## 4. Impact of COX-2 Inhibitors on Clinical Management of Arthritis

It is a widespread postulation that all NSAIDs, including COX-2 inhibitors, should be avoided, wherever possible, in patients with high risk of GI complications. The choice of NSAID remains confusing and controversial in the clinical management of arthritis. A common accord on the choice of drug in clinical management of arthritis is the risk benefit ratio. 

Any patient requiring chronic NSAID treatment for the management of arthritis may benefit from the COX-2 therapy. Patients who are at a high risk of GI bleeding, have a history of intolerance to traditional NSAIDs, or are not responding to traditional NSAIDs may be appropriate candidates for treatment with COX-2 selective inhibitors. These include the elderly, those with documented prior ulcers, and patients on concomitant steroids. In patients who are not at a high risk of serious GI events, cost should be considered as an important factor while considering the treatment options. Physicians should exercise extra caution when prescribing COX-2 inhibitors to patients with risk factors for heart disease. Patients with established ischemic heart disease, peripheral arterial disease, or cerebrovascular disease should be switched to traditional NSAIDs and gastroprotective agents should be considered in this situation. COX-2 inhibitors may be preferable in patients taking low-dose aspirin, since they do not interfere with platelet inhibition by aspirin 

## 5. Conclusion

Extensive evaluation of the safety and efficacy of NSAIDs and selective COX-2 inhibitors has revealed that both drugs are equally effective and are associated with increased risk of GI, renal, and CV, adverse effects. Physicians have to revise their indications for both the traditional NSAIDs and the COX-2 inhibitors in the management of arthritis and to give considerable attention to the balance of benefits and risks. Thorough assessment of patient's comorbid conditions and concomitant medications, along with precise monitoring during therapy, may help to decrease the threat of toxicity induced by traditional NSAIDs and selective COX-2 inhibitors. 

## References

[1] Bertolini A, Ottani A, Sandrini M (2002). Selective COX-2 inhibitors and dual acting anti-inflammatory drugs: critical remarks. *Current Medicinal Chemistry*.

[2] Hinz B, Brune K (2002). Cyclooxygenase-2—10 Years later. *Journal of Pharmacology and Experimental Therapeutics*.

[3] Martel-Pelletier J, Lajeunesse D, Reboul P, Pelletier JP (2003). Therapeutic role of dual inhibitors of 5-LOX and COX, selective and non-selective non-steroidal anti-inflammatory drugs. *Annals of the Rheumatic Diseases*.

[4] Parente L (2001). Pros and cons of selective inhibition of cyclooxygenase-2 versus dual lipoxygenase/cyclooxygenase inhibition: is two better than One?. *Journal of Rheumatology*.

[5] Altman RD, Hochberg MC, Moskowitz RW, Schnitzer TJ (2000). Recommendations for the medical management of osteoarthritis of the hip and knee: 2000 update. *Arthritis and Rheumatism*.

[6] Jordan KM, Arden NK, Doherty M (2003). EULAR Recommendations 2003: an evidence based approach to the management of knee osteoarthritis: report of a Task Force of the Standing Committee for International Clinical Studies Including Therapeutic Trials (ESCISIT). *Annals of the Rheumatic Diseases*.

[7] Hochberg MC (2002). New directions in symptomatic therapy for patients with osteoarthritis and rheumatoid arthritis. *Seminars in Arthritis and Rheumatism*.

[8] Green GA (2001). Understanding NSAIDs: from aspirin to COX-2. *Clinical Cornerstone*.

[9] Whittle BJR (2003). Gastrointestinal effects of nonsteroidal anti-inflammatory drugs. *Fundamental and Clinical Pharmacology*.

[10] Vane JR (1971). Inhibition of prostaglandin synthesis as a mechanism of action for aspirin-like drugs. *Nature: New biology*.

[11] Rainsford KD (1999). Profile and mechanisms of gastrointestinal and other side effects of nonsteroidal anti-inflammatory drugs (NSAIDs). *American Journal of Medicine*.

[12] Allison MC, Howatson AG, Torrance CJ, Lee FD, Russell RI (1992). Gastrointestinal damage associated with the use of nonsteroidal antiinflammatory drugs. *New England Journal of Medicine*.

[13] García Rodríguez LA, Cattaruzzi C, Troncon MG, Agostinis L (1998). Risk of hospitalization for upper gastrointestinal tract bleeding associated with ketorolac, other nonsteroidal anti-inflammatory drugs, calcium antagonists, and other antihypertensive drugs. *Archives of Internal Medicine*.

[14] García Rodríguez LA, Jick H (1994). Risk of upper gastrointestinal bleeding and perforation associated with individual non-steroidal anti-inflammatory drugs. *Lancet*.

[15] Langman MJS, Weil J, Wainwright P (1994). Risks of bleeding peptic ulcer associate with individual non-steroidal anti-inflammatory drugs. *Lancet*.

[16] Wolfe MM, Lichtenstein DR, Singh G (1999). Gastrointestinal toxicity of nonsteroidal antiinflammatory drugs. *New England Journal of Medicine*.

[17] Seager JM, Hawkey CJ (2001). ABC of the upper gastrointestinal tract: indigestion and non-steroidal anti-inflammatory drugs. *British Medical Journal*.

[18] Laine L (2002). The gastrointestinal effects of nonselective NSAIDs and COX-2-selective inhibitors. *Seminars in Arthritis and Rheumatism*.

[19] Bombardier C (2002). An evidence-based evaluation of the gastrointestinal safety of coxibs. *American Journal of Cardiology*.

[20] Grosser T, Fries S, FitzGerald GA (2006). Biological basis for the cardiovascular consequences of COX-2 inhibition: Therapeutic challenges and opportunities. *Journal of Clinical Investigation*.

[21] Hawkey CJ (1999). COX-2 inhibitors. *Lancet*.

[22] Masferrer JL, Zweifel BS, Manning PT (1994). Selective inhibition of inducible cyclooxygenase 2 in vivo is antiinflammatory and nonulcerogenic. *Proceedings of the National Academy of Sciences of the United States of America*.

[23] Micklewright R, Lane S, Linley W, McQuade C, Thompson F, Maskrey N (2003). Review article: NSAIDs, gastroprotection and cyclo-oxygenase-II-selective inhibitors. *Alimentary Pharmacology and Therapeutics*.

[24] British National Formulary

[25] Hawkey CJ (1997). The gastroenterologist’s caseload: contribution of the rheumatologist. *Seminars in Arthritis and Rheumatism*.

[26] Laine L, Smith R, Min K, Chen C, Dubois RW (2006). Systematic review: the lower gastrointestinal adverse effects of non-steroidal anti-inflammatory drugs. *Alimentary Pharmacology and Therapeutics*.

[27] Deeks JJ, Smith LA, Bradley MD (2002). Efficacy, tolerability, and upper gastrointestinal safety of celecoxib for treatment of osteoarthritis and rheumatoid arthritis: systematic review of randomised controlled trials. *British Medical Journal*.

[28] Wober W (1999). Comparative efficacy and safety of nimesulide and diclofenac in patients with acute shoulder, and a meta-analysis of controlled studies with nimesulide. *Rheumatology*.

[29] Lucker PW, Pawlowski C, Friederich I, Faiella F, Magni E (1994). Double-blind, randomised, multi-centre clinical study evaluating the efficacy and tolerability of nimesulide in comparison with etodalac in patients suffering from osteoarthritis of the knee. *European Journal of Rheumatology and Inflammation*.

[30] Schnitzer TJ, Constantine G (1997). Etodolac (Lodine) in the treatment of osteoarthritis: recent studies. *Journal of Rheumatology*.

[31] Neustadt DH (1997). Double blind evaluation of the long-term effects of etodolac versus ibuprofen in patients with rheumatoid arthritis. *Journal of Rheumatology. Supplement*.

[32] Silverstein FE, Faich G, Goldstein JL (2000). Gastrointestinal toxicity with Celecoxib vs nonsteroidal anti-inflammatory drugs for osteoarthritis and reumatoid arthritis: the CLASS study: a randomized controlled trial. *Journal of the American Medical Association*.

[33] Bombardier C, Laine L, Reicin A (2000). Comparison of upper gastrointestinal toxicity of rofecoxib and naproxen in patients with rheumatoid arthritis. *New England Journal of Medicine*.

[34] Schnitzer TJ, Burmester GR, Mysler E (2004). Comparison of lumiracoxib with naproxen and ibuprofen in the Therapeutic Arthritis Research and Gastrointestinal Event Trial (TARGET), reduction in ulcer complications: Randomised controlled trial. *Lancet*.

[35] Harris RC (2002). Cyclooxygenase-2 inhibition and renal physiology. *American Journal of Cardiology*.

[36] Komers R, Anderson S, Epstein M (2001). Renal and cardiovascular effects of selective cyclooxygenase-2 inhibitors. *American Journal of Kidney Diseases*.

[37] Patrignani P, Sciulli MG, Manarini S, Santini G, Cerletti C, Evangelista V (1999). COX-2 is not involved in thromboxane biosynthesis by activated human platelets. *Journal of Physiology and Pharmacology*.

[38] Catella-Lawson F, Crofford LJ (2001). Cyclooxygenase inhibition and thrombogenicity. *American Journal of Medicine*.

[39] Combe B, Swergold G, McLay J (2009). Cardiovascular safety and gastrointestinal tolerability of etoricoxib vs diclofenac in a randomized controlled clinical trial (The MEDAL study). *Rheumatology*.

[40] Kearney PM, Baigent C, Godwin J, Halls H, Emberson JR, Patrono C (2006). Do selective cyclo-oxygenase-2 inhibitors and traditional non-steroidal anti-inflammatory drugs increase the risk of atherothrombosis? Meta-analysis of randomised trials. *British Medical Journal*.

[41] Walter MF, Jacob RF, Day CA, Dahlborg R, Weng Y, Mason RP (2004). Sulfone COX-2 inhibitors increase susceptibility of human LDL and plasma to oxidative modification: comparison to sulfonamide COX-2 inhibitors and NSAIDs. *Atherosclerosis*.

[42] Solomon DH, Schneeweiss S, Glynn RJ (2004). Relationship between selective cyclooxygenase-2 inhibitors and acute myocardial infarction in older adults. *Circulation*.

[43] Garner SE, Fidan DD, Frankish R, Maxwell L (2005). Rofecoxib for osteoarthritis. *Cochrane Database of Systematic Reviews*.

[44] Garner S, Fidan D, Frankish R (2002). Celecoxib for rheumatoid arthritis. *Cochrane Database of Systematic Reviews*.

[45] Edwards JE, McQuay HJ, Moore RA (2004). Efficacy and safety of valdecoxib for treatment of osteoarthritis and rheumatoid arthritis: systematic review of randomised controlled trials. *Pain*.

[46] Zacher J, Feldman D, Gerli R (2003). A comparison of the therapeutic efficacy and tolerability of etoricoxib and diclofenac in patients with osteoarthritis. *Current Medical Research and Opinion*.

